# Quantitative Magnetic Resonance Imaging of Femoral Head Articular Cartilage Change in Patients with Hip Osteonecrosis Treated with Extracorporeal Shock Wave Therapy

**DOI:** 10.1155/2022/8609868

**Published:** 2022-06-13

**Authors:** Lijun Shi, Xu Yang, Peixu Wang, Xiangwei Ma, Dan Li, Xinjie Wu, Fuqiang Gao, Wei Sun

**Affiliations:** ^1^Department of Orthopedic Surgery, The First Affiliated Hospital of Zhengzhou University, Zhengzhou, Henan, China; ^2^Department of Orthopedics, Peking University China-Japan Friendship Clinical Hospital, Beijing 100029, China; ^3^Department of Orthopedic Surgery, Centre for Osteonecrosis and Joint-Preserving & Reconstruction, China-Japan Friendship Hospital, Beijing 100029, China; ^4^Department of Rehabilitation Medicine, China-Japan Friendship Hospital, Beijing 100029, China; ^5^Department of Pediatrics, The First Affiliated Hospital of Zhengzhou University, Zhengzhou 450000, Henan, China; ^6^Department of Molecular Medicine and Surgery, Karolinska Institute, Stockholm 171 76, Sweden

## Abstract

**Background:**

Multiple reports have demonstrated the therapeutic potential of extracorporeal shock wave (ESWT) in osteonecrosis of the femoral head (ONFH). However, few studies reported the changes in hip articular cartilage after the intervention. This study aimed to investigate the effect of ESWT on femoral head cartilage using a novel technique, quantitative T2-mapping magnetic resonance imaging.

**Methods:**

A total of 143 eligible patients with unilateral early-stage ONFH were randomized into the ESWT group and control group. Seventy-three patients in the ESWT group received two sessions of ESWT with oral drug treatment, while seventy patients in the control group received oral drug treatment only. The visual analog pain scale (VAS) and Harris hip score (HHS) at 3-month, 6-month, and 12-month follow-up were used as the clinical evaluation index. The radiological evaluation index used the T2 mapping values, necrotic size, and China-Japan Friendship Hospital (CJFH) classification.

**Results:**

A total of 143 patients (62 females and 81 males) were finally included, and the characteristics before treatment were comparable between the two groups. At the last follow-up (12 months), the T2 values and ΔT2 changes in the ESWT group were all smaller than those in the control group (*p*=0.042; *p*=0.039), while the CJFH classification of ONFH and necrotic lesion size were not statistically significant. At 3 months and 6 months, the VAS in the ESWT group was lower than that in the control group (*p*=0.021; *p*=0.046) and the HHS in the ESWT group was higher (*p*=0.028; *p*=0.039). However, there were no significant differences in the VAS and HHS at 12 months between the ESWT and control groups.

**Conclusions:**

The results of the current study indicated that, based on drug treatment, ESWT is an effective treatment method for nontraumatic ONFH, which could result in significant pain relief and function restoration. Furthermore, it could delay the injury of femoral head cartilage during the progression of ONFH.

## 1. Introduction

Osteonecrosis of the femoral head (ONFH) is a major side effect related to high-dose corticosteroid administration, which occurs frequently in relatively young adults (age, 30–50 years) [1, 2]. It is a progressive pathological condition characterized by large amounts of death of bone cells and tissue necrosis due to insufficient circulation, leading to femoral head collapse and secondary hip osteoarthritis. Most patients, if left not treated, may require total hip arthroplasty (THA) in the early stage. Postcollapse ONFH has been one of the most common reasons for primary THA in many countries. Considering that ONFH typically affects the younger population, preventing or delaying the time of femoral head collapse is the primary goal of the intervention.

Currently, the optimal therapy options for ONFH remain controversial both surgical and nonsurgical options have been reported with variable levels of success. These procedures aim to preserve the femoral head and facilitate necrotic tissue regeneration. Early intervention in the precollapse stage of the femoral head is still critical for a successful outcome [3–5]. High-energy extracorporeal shock wave therapy (ESWT) is a noninvasive technology that has been successfully used in various musculoskeletal conditions, such as tennis elbow, plantar fasciitis, fracture nonunions, and lateral epicondylitis [6–8]. The extracorporeal shock wave is a kind of mechanical wave with high pressure and energy that forms reflection and precipitation on the interface between soft tissue and bone [9–11]. Multiple clinical studies have demonstrated the therapeutic potential of ESWT in the early stage of ONFH with improved clinical prognosis and a decrease in osteonecrosis [12, 13]. Though the exact mechanisms remain unknown, studies reported that ESWT may promote the expression of bone growth factors and angiogenic growth factors [14], leading to neovascularization and osteogenesis. ESWT can activate many cellular processes critical to the above biological processes [15–17].

MRI remains the most important aid for the diagnosis of ONFH, with a sensitivity of 99% and a specificity of 98% [18]. However, conventional MRI has limited efficacy for cartilage evaluation and cannot detect lesions in the early stages. T2 (transverse relaxation time) mapping is a novel technique for the compositional assessment of the articular cartilage using MRI [19]. Studies have shown that T2 mapping could be used as a useful predictor of cartilage degeneration of the hip joint as well as the knee joint [20, 21]. Increased T2 values are thought to be a marker of cartilage degeneration.

T2 relaxation time is sensitive to tissue hydration and biochemical components and is an important noninvasive marker of cartilage damage [22]. Under normal conditions, the synovial fluid near the articular cartilage is hyperintense on T2-weighted images due to the presence of water protons. In cartilage, however, water protons are immobilized by the collagen-proteoglycan matrix, resulting in attenuation of T2 values (low signal intensity). The femoral head collapses in the later stage of ONFH and damages the cartilage, resulting in a decrease in bone quality in the cartilage, making this signal difference more pronounced. Bone quality is a collective term referring to the mechanical properties, architecture (thickness of cortical bone and distribution of trabecular network), degree of mineralization of the bone matrix, and chemistry as well as the remodeling properties of bone. Therefore, the T2 relaxation time estimates based on MRI that showed a strong correlation with bone strengths in cartilage have been associated with matrix damage.

Early detection of cartilage degeneration is important for predicting the subsequent natural history and determining the appropriate timing for surgery. In the process of ONFH, articular cartilage degeneration appears to be a secondary effect of mechanical stress following articular collapse [23]. It has been speculated that the femoral head articular cartilage is unaffected in patients with noncollapsed osteonecrosis [24]. On the other hand, degeneration of articular cartilage was revealed in patients with noncollapsed osteonecrosis using T2 mapping [25]. So far, the majority of studies about ESWT in ONFH focused on necrotic bone repair; however, few studies reported its effect on cartilage metabolism after treatment.

Early detection of cartilage degeneration can help to improve a patient's outcome. No information is currently available on the application of T2 mapping to examine cartilage matrix changes in ONFH. The primary purpose of this study was to evaluate cartilage conditions in early-stage ONFH patients after ESWT therapy using T2 mapping. The secondary purpose was to assess the functional outcomes of ESWT in the treatment of ONFH.

## 2. Materials and Methods

### 2.1. Study Subjects

This study has been approved by the Ethics Committee of our hospital (2018-109-K78) and has completed clinical registration in the “China Clinical Experiment Registration Center” recognized by WHO. The approved clinical registration number is ChiCTR1800016306.

Through TV campaigns, advertisements, and physician (department of hematology, rheumatology, nephrology, and radiology) referrals, we included patients with ONFH that were confirmed at the screening by radiographs and MRI. In T1-weighted sequences, there is a low signal line between healthy bone and ischemic bone, known as a “band-like lesion,” which corresponds to a zone of sclerosis and fibrosis. T2-weighted sequences show evidence of a second inner line of high signal (double line sign), indicative of hypervascularity resulting from the repair process [26].

**Figure 1:**
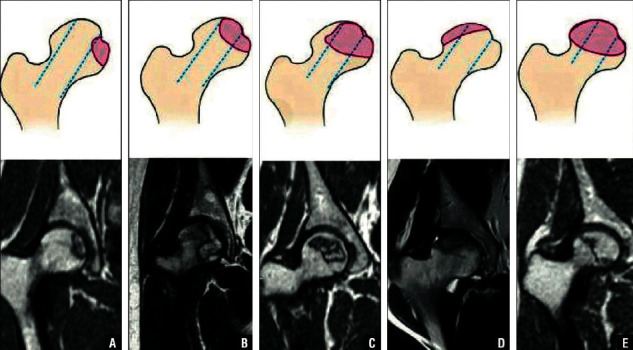
Schematic diagrams (top) and magnetic resonance images (bottom) of the China-Japan Friendship Hospital classification of osteonecrosis of the femoral head based on the 3 pillars. Type M: necrosis involves the medial pillar (a). Type C: necrosis involves the medial and central pillars (b). Type L1: necrosis involves all 3 pillars, but the lateral pillar is partially preserved (c). Type L2: necrosis involves the entire lateral pillar and part of the central pillar (d). Type L3: necrosis involves all 3 pillars, including the cortical bone and marrow (e).

All patients provided written informed consent before participating in the prospective trial, and prior approval for this study was obtained from the Scientific Review Board of China-Japan Friendship Hospital. MRI examination was of high susceptibility (>90%) and specificity (>95%), and it has been used as gold criteria for the diagnosis of ONFH. Each hip's ONFH was staged according to the Association Research Circulation Osseous (ARCO) [27] and classified according to the China-Japan Friendship Hospital (CJFH) classification system as M, C, L1, L2, and L3 [28] (Figure 1). The location of the necrotic lesion in the femoral head was considered the basis of classification.

The patients enrolled in the study were required to meet the inclusion criteria for participation: (1) aged 18–65 years; (2) unilateral ONFH; (3) early ONFH of stage I, II, or IIIa according to ARCO; (4) steroid-induced ONFH. Patients were excluded in the case of the following exclusion criteria: (1) poor physical condition; (2) bilateral ONFH; (3) late ONFH of stage IIIb, IIIC, or IV according to ARCO; (4) idiopathic ONFH or ONFH caused by other reasons, such as heavy drinking, trauma, and decompression sickness except for steroid; (5) easy bleeding tendencies or blood coagulation disorders; (6) known contraindication to MRI examination, including claustrophobia and metal intrauterine devices implantation; (7) contraindications to ESWT: subjects with a heart pacemaker, heart stent implantation, hemorrhagic disease, cancer, and thrombus formation; and (8) skin ulceration and various infections of treated areas, acute and massive joint effusion, bone infections, and large bone defect (>1 cm).

### 2.2. Study Design

This prospective, randomized, parallel, controlled study was conducted at our single institution from August 2018 to May 2021. After screening according to the inclusion and exclusion criteria, the eligible patients were allocated into two groups using computer-generated random assignment concealment using sealed envelopes. Both the ESWT group (*n* = 75) and control group (*n* = 77) patients were administered with oral herbal Fufang Xian Ling Gu Bao (Main ingredient: Icariin, Tongjitang (Guizhou) Pharmaceutical Co., Ltd. of Sinopharm Group) (three capsules, p.o. b.i.d.) and alendronate sodium tablets (70 mg p.o. q.w., Merck & Co., Inc., Peking) orally for six weeks. Both drugs have been approved by FDA for the treatment of ONFH. Meanwhile, the ESWT group also received two sessions of ESWT on the sick hip, and the interval was one week. All the patients were maintained at 50% weight-bearing with two crutches for 6 weeks after starting treatment.

### 2.3. Extracorporeal Shock Wave Treatment

**Figure 2:**
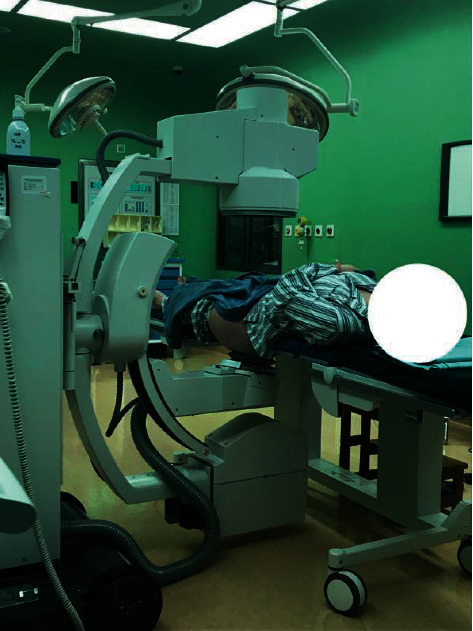
Operation diagram of an extracorporeal shock wave for ONFH.

Electromagnetic Shock Wave Emitter (Dornier Compact DELTA II, Germany) was used for the shock wave treatment; the penetration depth and focus diameter were 15 and 4 mm, respectively. The patients were positioned in the bed with sick hip joints in an external rotation position (Figure 2). Subsequently, the coupling gel was smeared uniformly at the interface between the head of the device and the skin to reduce the loss of shock wave energy. In this study, we selected 4–6 target sites on the femoral head as the central points of shock waves under radiographical guidance, and every target site got 400–600 shots, avoiding the proximal blood vessels and spinal nerve trunk during the procedure. The energy flux density per shot was more than 0.44 mJ/mm^2^, and the frequency of pulse frequency was set at 2-3 Hz in a single session. All the ESWT procedures were performed or supervised by the principal investigator. Local complications such as the formation of hematoma, petechial hemorrhage, swelling, deep vein thrombosis, and superficial infection were all recorded. After the shock wave treatment, the patients were instructed to walk on crutches with nonweight-bearing on the affected extremity for 6 weeks.

### 2.4. T2-Mapping MRI Examination

All MRI scans were performed on a 3T MRI unit (GE DISCOVERY MR750, China-Japan Friendship Hospital) with a surface coil wrapped around the hips (SENSE Body Coil, Philips Healthcare, The Netherlands). The patients were imaged in a supine position, with the hips positioned neutrally and legs straight. Imaging sequences included fat-saturated proton-density weighted imaging (PD-FS), three-dimension double echo steady-state sequence (3D-DESS), and quantitative Cube T2 -mapping. The MRI parameters were optimized to achieve the highest signal noise ratio (SNR) and image quality. T2-mapping was conducted using similar imaging parameters to enable comparison. The repetition time (TR) was 1000 ms, while the echo time (TE) was set to 32 ms. The slice thickness was chosen to be 3.5 mm with a spacing of 0.7 mm. The field of view (FOV) was 256 mm × 256 mm. And the number of excitations (NEX) was set to 1. Each participant was required to rest for 30 minutes before the knee scan to ensure that the cartilage was resting.

We used an advanced cartilage analysis application (IntelliSpace Portal, Philips Healthcare) to analyze the reconstructed T2 maps. Local T2 values can be determined through T2 mapping using multiecho (ME) SE methods [29].

### 2.5. Assessment

To evaluate the clinical efficacy and safety of invasive ESWT in hip articular cartilaginous metabolism, all patients in both groups were prospectively followed-up clinically and radiographically at the baseline and then at 3, 6, and 12 months after treatment initiation. The clinical evaluation parameters included clinical assessment of pain using a visual analog scale (VAS), where 0 indicated no pain and 10 indicated severe pain, and assessment of function, activity, and motion of the hip using the Harris hip score (HHS) [30].

In the radiological evaluation, the femoral cartilage was evenly divided into 12 parts radially, and the acetabular cartilage was evenly divided into 6 parts radially. Furthermore, the cartilage is evenly divided into two layers parallel to the surface. Three authors (Lijun Shi, Peixu Wang, and Wei Sun) independently reviewed the images of the T2-mapping MRI examination and qualitatively assessed the hip articular cartilage injury, determining whether the treatment could reverse the cartilage injury. For T2 measurements, the region of interest (ROI) was drawn over the entire area of each segment and layer. At the same time, the value of the T2 score obtained from the T2 mapping MRI image, the location and size of the necrotic lesion, and the CJFH type of ONFH based on the image were also recorded for quantitative evaluation.

### 2.6. Statistical Analysis

All data analyses were performed using the SPSS version 22.0.0 software (SPSS, Chicago, IL, USA). The quantitative variables from baseline to 12 months, including the age of patients, T2 value, size of the necrotic lesion, VAS, and HHS, were represented as the mean ± standard deviation (SD), and the between-group difference was evaluated by ANOVA and post hoc tests. The between-group difference of dichotomous data, such as gender, sick side, ARCO stage, and CJFH classification type, was evaluated by a continuous correction chi-square test. *P* values < 0.05 were considered statistically significant.

## 3. Results

Between 2018 and 2021, 156 ONFH patients with ONFH met our inclusion criteria; of those, 2 cases of bilateral ONFH, 1 case of alcohol-induced ONFH, and 1 case of idiopathic ONFH were excluded according to the exclusion criteria. During the study, another 2 cases and 7 cases in the ESWT group and control group were lost to follow-up, respectively. Thus, a total of 143 patients were finally included.  Table 1 shows the baseline clinical status of the patients. Patients in both the ESWT group (*n* = 73) and the control group (*n* = 70) were similar in the demographic and clinical characteristics at the initiation of the clinical trial, concerning gender, age, side, VAS, HHS, ARCO stage, CJFH type, the baseline value of T2, and the size of necrotic lesion of the femoral head before treatment (all *p* > 0.05).

### 3.1. Radiological Results

Table 2 shows the *T*2 changes in hip articular cartilage before and after treatment. There was no significant difference in *T*2 value before treatment in the two groups of patients. At the last follow-up (12 months), the injury of cartilage was aggravated in both groups with the *T*2 value being increased. The mean *T*2 value increased from 44.58 ± 4.99 to 49.50 ± 4.94 in the ESWT group, which was lower than that (52.99 ± 7.18) in the control group (*t* = 0.357; *p*=0.042). The mean changes in *T*2 values in the ESWT group (4.42 ± 7.04) were also lower than those in the control group (7.85 ± 6.68) (*t* = −0.153; *p*=0.039). These results demonstrated that, in patients with ONFH, the degeneration of hip articular cartilage was still in process under treatment. However, based on drug treatment, adding ESWT was more effective in holding degeneration of cartilage than single drug administration alone.

Table 3 shows the CJFH type changes before and after treatment in both ESWT and control groups. There was no significant difference in the number of each CJFH subtype (namely, *M* + *C*, *L*1, *L*2, and *L*3) between the two groups before treatment (*χ*^2^ = 1.875; *p*=0.599). At 12 months after the initial treatment, the cases of *M* + *C* type and *L*1 type increased, whereas the cases of *L*2 type and *L*3 type decreased in both two groups, indicating a concentric bone repairment occurrence. There were 26 cases of *M* + *C* type, 29 cases of *L*1 type, 8 cases of *L*2 type, and 10 cases of *L*3 type in the ESWT group; there were 25 cases of *M* + *C* type, 26 cases of *L*1 type, 6 cases of *L*2 type, and 13 cases of *L*3 type in the control group. Though the subtype difference was not significant between the two groups (*χ*^2^ = 0.623; *p*=0.891), the cases of *M* + *C* type and *L*1 type in the ESWT group were more than those in the control groups. These results showed that both different treatment methods in the two groups had a positive effect on the repairment of necrotic lesions, respectively, and the effect of ESWT plus drug treatment on repairment was more apparent.

Table 4 shows the size changes of the necrotic lesion in the femoral head before and after treatment. The baseline data of necrotic size in the ESWT group (27.1 ± 4.6 mm^2^) and the control group (27.6 ± 4.0 mm^2^) were comparable (*p*=0.472). After 12 months, the mean necrotic size in the ESWT group (18.7 ± 7.5 mm^2^) was nearly smaller than that in the control group (20.7 ± 7.3 mm^2^), although the difference fell short of statistical significance (*p*=0.051). The mean necrotic size reduced by 8.4 ± 7.4 mm^2^ and 6.9 ± 9.0 mm^2^ in the ESWT and control groups after treatment, respectively, and the difference was not statistically significant (*t* = −0.336; *p*=0.056). The above results indicated that ESWT was an important adjunct to drug treatment for ONFH, which could effectively reduce the necrotic lesion of the femoral head. However, the advantages of ESWT for ONFH treatment were not obvious according to statistical analysis. Maybe it was because the size of the necrotic lesion was relatively larger (27.1 ± 4.6 mm^2^) in the ESWT group, and there were more ARCO IIIA patients (64/143, 44.8%).

### 3.2. Clinical Results

#### 3.2.1. VAS Score

**Figure 3:**
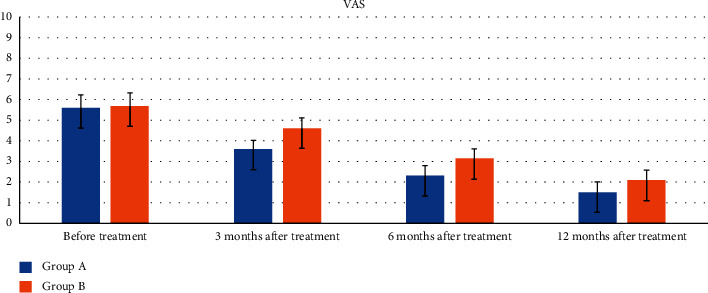
Histogram of VAS follow-up comparison between the two groups before and after treatment (ESWT group; control group).

Table 5 shows the changes in VAS before and after treatment. Before treatment, there was no difference in VAS between the two groups (*p* > 0.05). At the last follow-up, all patients in both groups had significant relief of hip pain with VAS reduction according to within-group analysis (all *p* < 0.05) (Table 5; Figure 3). At 3 and 6 months after treatment, the VAS in the ESWT group was lower than that in the control group (all *p* < 0.05). However, the VAS in the ESWT and control groups was comparable at 12 months (*t* = 0.494; *p*=0.078), indicating that there was no difference in hip pain relief between the two groups.

#### 3.2.2. HHS Score

**Figure 4:**
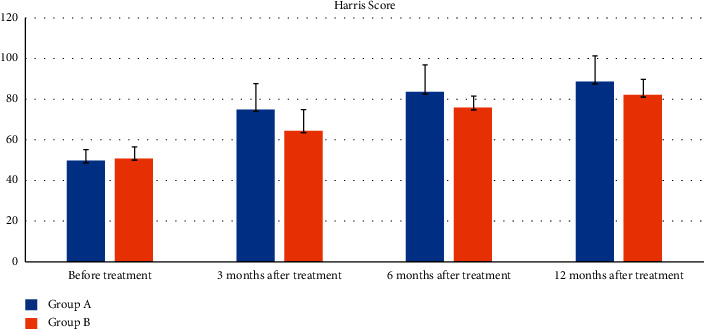
Follow-up comparison histogram of HHS between two groups before and after treatment (ESWT group; control group).

Table 6 shows the changes in HHS before and after treatment. The hip function changes were consistent with the hip pain changes. Before treatment, there was no difference in HHS between the two groups (*p*=0.283). At the last follow-up, all patients in both groups had significant hip function improvement with a rise in HHS according to within-group analysis (all *p* < 0.05) (Table 6; Figure 4). At 3 and 6 months after treatment, the HHS in the ESWT group was higher than that in the control group (all *p* < 0.05). However, the HHS in the ESWT and control groups was comparable at 12 months (*t* = −0.879; *p*=0.381), indicating there was no difference in hip function improvement between the two groups.

### 3.3. Side Effects

No acute or major ESWT-related complications or adverse drug reactions occurred during the study in ESWT or control group, while 11 cases appeared with slight skin swelling and petechiae after ESWT. However, these symptoms could disappear spontaneously within 1 week without special intervention, and patients had no uncomfortable feelings. At 12 months after initial treatment, no patients showed the progression of necrotic lesion or collapse (>2 mm) of the femoral head, and no patients needed total hip arthroplasty surgery.

## 4. Discussion

The purpose of this present study was to evaluate radiological and clinical outcomes of ESWT for the treatment of early-stage ONFH. During the follow-up, we observed hip pain and function changes, necrotic lesion size changes, and the CJFH classification changes of ONFH before and after treatment. Furthermore, we also observed the hip articular cartilage abnormalities using MRI T2 mapping, which was the novelty of this research. Compared with the control group individuals, who received oral drug treatment only, we found out that the hip pain relief and hip function improvement at 3 and 6 months after intervention in the ESWT group were more remarkable. However, no statistically significant difference was observed at 12 months of follow-up, indicating that ESWT was more effective in clinical promotion in a short period (<6 months) after initial treatment. In radiological evaluation, the T2 relaxation values of the cartilage layer of the femoral head in the ESWT group were significantly lower than those in the control group at the last follow-up, and the necrotic lesion size was also nearly smaller in the ESWT group. These radiological results illustrated that ESWT with drug treatment could effectively promote bone regeneration of necrotic lesions and delay the degeneration of femoral head cartilage.

ESWT has shown significant beneficial advantages in multiple musculoskeletal diseases. The results of this study are similar to previous studies. Algarni and Al Moallem thought that ESWT may halt or delay the radiographic progression of ONFH in the precollapse stage [31]. Vulpiani et al. conducted similar clinical research with long-term follow-up (>24 months) and concluded that ESWT could help to prevent the progression of the area of necrotic necrosis and manage pain [32]. Several systematic reviews or meta-analyses also confirmed its therapeutic potential and significant advantages in ONFH [33–36]. Extracorporeal shock waves are acoustic waves of extremely high pressure and velocity. Shock waves can travel through fluid and soft tissue, and their effects occur at sites where there is a change in impedance, such as the bone-soft tissue interface. When shock waves are directed at the bone, multiple interfaces between soft tissue and bone result in the reflection and deposition of shock wave energy [37]. This deposition may be responsible for the osteogenesis and angiogenesis effects of this therapy seen in some studies. However, few studies reported the effect of ESWT on cartilage.

The collapse of the femoral head is a major turning point for the progression of ONFH, and cartilage injury plays an important role in the collapse. The development of MRI technology provides a tool for the early diagnosis of irreversible articular cartilage damage, which is of great significance in the treatment of cartilage-related diseases [38–40]. *T*2 mapping quantitative MRI is a novel technology for cartilage examination that can help diagnose early degeneration of cartilage preceding visible damage [20]. The *T*2 relaxation values are closely related to hydration and collagen fiber integrity, water content, and external pressure. Focal increases in T2 relaxation time estimates based on MRI that showed a strong correlation with bone quality in cartilage have been associated with matrix damage, in particular with a loss of collagen integrity and an increase in water content [22]. *T*2 mapping for cartilage assessment has been successfully applied in numerous clinical studies. Most of these studies were carried out in patients with osteoarthritis or patients after cartilage repair [41, 42]. Bittersohl et al. reported the first study on *T*2 mapping of the hip joint in 2009 [43]. However, this study has its limitations because the MRI used is a 1.5-T system, where image resolution was not enough to differentiate between acetabular and femoral cartilage. In our current study, we used a 3.0-T system to recognize the differentiation and delineation of acetabular and the femoral head cartilage. *T*2 mapping demonstrated higher values in femoral head cartilage in the control group, indicating a more severe injury of the hip cartilage.

Notably, there are some potential limitations to this study. First, the primary purpose was to assess the hip cartilage changes, but the time of follow-up was relatively short (not more than 12 months), lacking a long-term evaluation. However, at 12 months, the clinical index has reached a relatively stable level. Second, the ONFH in the patients included in the study was caused by corticosteroids, but we did not quantitatively calculate the total dose that patients had used. Third, the effect of ESWT on the cartilage can only be partial, and we did not take into account the effects of another factor that could influence the metabolism of bone and cartilage. Furthermore, our study lacked comparisons of interindividual and interregional differences in T2 values.

## 5. Conclusion

Based on the drug treatment for ONFH, ESWT could promote bone repair and decrease the necrotic lesion of the femoral head. And ESWT may delay the injury by improving cartilaginous metabolism effectively with reliable safety. Therefore, ESWT helps ease the hip pain symptom and improve hip function for the early stage of steroid-induced ONFH. These results also illustrate that ESWT could contribute to preventing osteoarthritis secondary to ONFH, providing evidence-based solutions for the application of ESWT in the prevention of secondary osteoarthritis after ONFH.

## Figures and Tables

**Table 1:** The baseline clinical status of the patients.

Variables	ESWT group (*n* = 73)	Control group (*n* = 70)	*χ* ^2^/*t*	*P*
*Gender (male/female)*	43/30	38/32	0.310	0.577
*Age (years)*	47.9 ± 15.3	46.0 ± 14.7	0.748	0.465
*Side (left/right)*	38/35	34/36	0.173	0.677
*T2 value (ms)*	44.58 ± 4.99	45.04 ± 4.34	0.589	0.557
*Necrotic size (mm* ^*2*^)	27.1 ± 4.6	27.6 ± 4.0	−0.722	0.472
*VAS*	5.6 ± 6.0	5.7 ± 6.3	0.763	0.447
*Harris*	49.8 ± 5.6	50.8 ± 5.6	−1.079	0.283
*CJFH type*				
*M* + *C* type	28	22	1.875	0.599
*L*1 type	18	24		
*L*2 type	14	11		
*L*3 type	13	13		
*ARCO stage*				
Stage I	5	4	0.813	0.666
Stage II	38	32		
Stage IIIA	30	34		

**Table 2:** *T*2 changes of hip articular cartilage before and after treatment in both groups.

Variables	ESWT group (*n* = 73)	Control group (*n* = 70)	*t*	*P*
*T*2 before treatment	44.58 ± 4.99	45.04 ± 4.34	0.589	0.557
*T*2 after treatment (12 months)	49.50 ± 4.94	52.99 ± 7.18	0.357	0.042
*T*2 changes (Δ)	4.42 ± 7.04	7.85 ± 6.68	−0.153	0.039

**Table 3:** CJFH type changes before and after treatment in both groups.

Variables	ESWT group (*n* = 73)	Control group (*n* = 70)	*χ* ^2^	*P*
*CJFH type before treatment*				
*M* + *C* type	28	22	1.875	0.599
*L*1 type	18	24		
*L*2 type	14	11		
*L*3 type	13	13		
*CJFH type after treatment*				
*M* + *C* type	26	25	0.623	0.891
*L*1 type	29	26		
*L*2 type	8	6		
*L*3 type	10	13		

**Table 4:** The size changes of necrotic lesions before and after treatment in both groups.

Variables	ESWT group (*n* = 73)	Control group (*n* = 70)	*t*	*P*
Size before treatment	27.1 ± 4.6	27.6 ± 4.0	−0.722	0.472
Size after treatment (12 months)	18.7 ± 7.5	20.7 ± 7.3	−0.050	0.051
Size changes (Δ)	8.4 ± 7.4	6.9 ± 9.0	−0.336	0.056

**Table 5:** The changes of VAS before and after treatment in both groups.

Variables	ESWT group (*n* = 73)	Control group (*n* = 70)	*t*	*P*
VAS before treatment	5.6 ± 0.6	5.7 ± 0.6	0.763	0.447
VAS at 3 months	3.6 ± 0.4	4.6 ± 0.5	−0.286	0.021
VAS at 6 months	2.3 ± 0.5	3.1 ± 0.5	0.363	0.046
VAS at 12 months	1.5 ± 0.5	2.1 ± 0.5	0.494	0.078

**Table 6:** The changes of HHS before and after treatment in both groups.

Variables	ESWT group (*n* = 73)	Control group (*n* = 70)	*t*	*P*
HHS before treatment	49.8 ± 5.6	50.8 ± 5.6	−1.079	0.283
HHS at 3 months	75.0 ± 12.7	64.6 ± 10.1	0.218	0.028
HHS at 6 months	83.5 ± 13.4	75.7 ± 5.6	−0.735	0.039
HHS at 12 months	88.7 ± 12.6	82.0 ± 7.7	−0.879	0.381

## Data Availability

The datasets used and/or analyzed during the current study are available from the corresponding author upon reasonable request.
